# Supplementary material for: CIRPIN: Learning Circular Permutation-Invariant Representations to Uncover Putative Protein Homologs
Source: bioRxiv. 2025 Nov 18:2025.11.18.689110. Preprint. [Version 1] doi: 10.1101/2025.11.18.689110 (PMC12667903; doi:10.1101/2025.11.18.689110)
Supplement: Supplement 1 [file NIHPP2025.11.18.689110v1-supplement-1.pdf]

bioRxiv preprint doi: https://doi.org/10.1101/2025.11.18.689110; this version posted November 18, 2025. The copyright holder for this preprint (which was not certified by peer review) is the author/funder, who has granted bioRxiv a license to display the preprint in perpetuity. It is made available under aCC-BY 4.0 International license.

A Supplementary Material

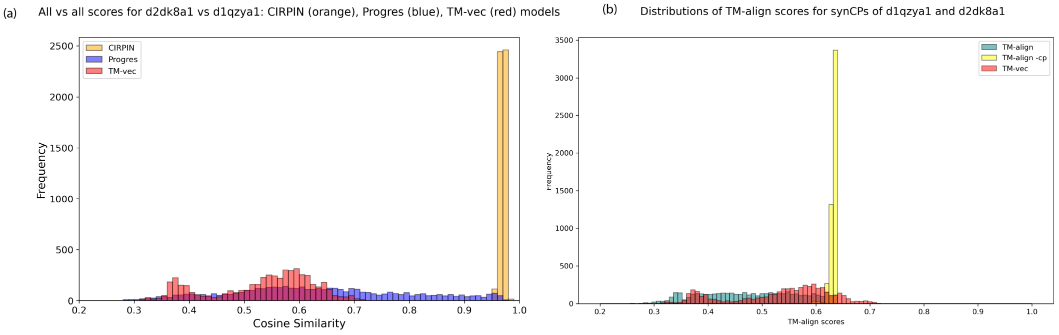

(c) Minimum synCP score

CP Pair	CIRPIN	Progres	TM-Vec
d1qasa2 & d1rsya1	0.975407	0.412015	0.658488
d1puja_ & d1zbda_	0.883974	0.342954	0.424968
d1cida1 & d1dcea2	0.809480	0.388181	0.392981
d1vhra_ & d1phra_	0.782694	0.364720	0.403653
d1mkza1 & d1jbwa1	0.742505	0.343785	0.398089
d2dk8a1 & d1qzya1	0.953684	0.267652	0.310037
d1iu9a_ & d1h0ra_	0.736251	0.379321	0.479205
d1qdma1 & d2gtga_	0.974847	0.674746	0.726851
d1booa_ & d1g38a1	0.847141	0.477426	0.469344
d3cnaa_ & d2pela_	0.989974	0.461548	0.632790
d1p8ja1 & d1ca1a2	0.974471	0.413952	0.442830

(d) Range of synCP score distributions

CP Pair	CIRPIN	Progres	TM-Vec
d1qasa2 & d1rsya1	0.019764	0.583527	0.227358
d1puja_ & d1zbda_	0.075348	0.630037	0.234344
d1cida1 & d1dcea2	0.070560	0.571518	0.281679
d1vhra_ & d1phra_	0.055206	0.572321	0.118635
d1mkza1 & d1jbwa1	0.093402	0.618927	0.161224
d2dk8a1 & d1qzya1	0.027627	0.705396	0.409501
d1iu9a_ & d1h0ra_	0.064763	0.581464	0.150846
d1qdma1 & d2gtga_	0.020889	0.322977	0.198557
d1booa_ & d1g38a1	0.067540	0.483792	0.246818
d3cnaa_ & d2pela_	0.009133	0.537570	0.334193
d1p8ja1 & d1ca1a2	0.013656	0.577414	0.225306

Supplementary Figure S1: (a-d) EDITComparison of Progres [9], CIRPIN, and TM-vec [10] performance on scoring a benchmark dataset of circular permutant pairs. (a) Representative distribution of synCP scores for circular permutants QZ and K8. (b) Minimum synCP scores taken from distributions shown in (a) for all pairs of circular permutants; higher scores (indicating structural similarity) are highlighted in yellow. (c) Range of synCP score distributions for Progres, CIRPIN, and TM-vec; smaller ranges (indicating greater invariance to circular permutation) are highlighted in yellow. Plotting the distributions across all synCP combinations reveals each model's sensitivity to positional reordering. We use the distribution range as a measure of invariance to circular permutation, with smaller ranges reflecting greater invariance, and also report the minimum score to capture cases where a model spuriously indicates dissimilarity due to reordering.

bioRxiv preprint doi: https://doi.org/10.1101/2025.11.18.689110; this version posted November 18, 2025. The copyright holder for this preprint (which was not certified by peer review) is the author/funder, who has granted bioRxiv a license to display the preprint in perpetuity. It is made available under aCC-BY 4.0 International license.

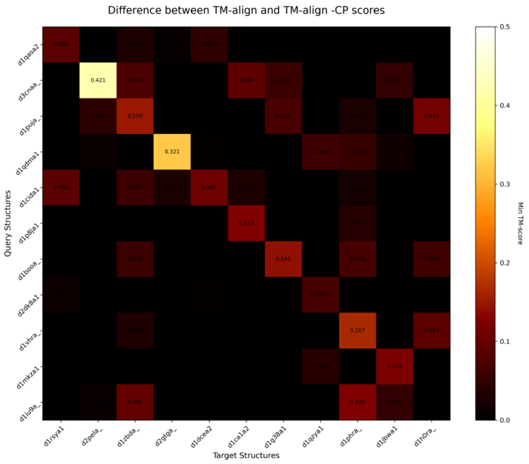

Supplementary Figure S2: ΔCP scores (difference between TM-align and TM-align -cp scores) for the test set of CPs.

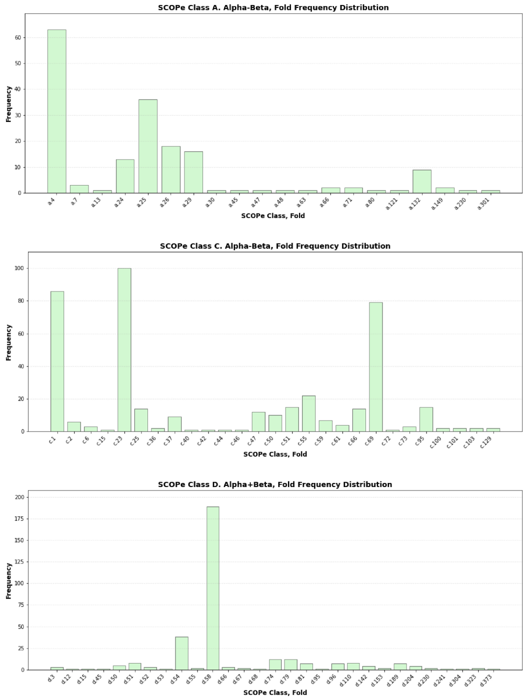

Supplementary Figure S3: Distribution of SCOPe CPs by SCOPe class and fold
